# Physical activity and sedentary behavior legislation in Canadian childcare facilities: an update

**DOI:** 10.1186/s12889-018-5292-1

**Published:** 2018-04-11

**Authors:** Leigh M. Vanderloo, Patricia Tucker

**Affiliations:** 1 Child Health and Evaluative Sciences, The Hospital for Sick Children, 686 Bay St, Toronto, ON M5G 0A4 Canada; 2 School of Occupational Therapy, Faculty of Health Sciences, University of Western Ontario, 1201 Western Road, Elborn College, Room 2547, London, ON N6G 1H1 Canada

## Abstract

**Background:**

Within the childcare sector, physical activity and sedentary behaviors are not legislated at a national level in Canada. Efforts have been undertaken to identify factors within childcare facilities which support and deter physical activity and sedentary behaviors. The purpose of this paper was to provide an amended review of the legislative landscape, at the provincial and territorial level, regarding physical activity and sedentary behaviors (via screen-viewing) in Canadian childcare centers.

**Methods:**

Individual childcare acts and regulations for each province and territory were collected; documents were reviewed with a focus on sections devoted to child health, physical activity, screen time, play, and outdoor time. An extraction table was used to facilitate systematic data retrieval and comparisons across provinces and territories.

**Results:**

Of the 13 provinces and territories, 8 (62%) have updated their childcare regulations in the past 5 years. All provinces provide general recommendations to afford gross motor movement; but the majority give no specific requirements for how much or at what intensity. Only 3 provinces (Northwest Territories, Nunavut, and Nova Scotia) explicitly mentioned daily physical activity while all provinces’ and territories’ required daily outdoor play. Only 1 province (New Brunswick) made mention of screen-viewing.

**Conclusions:**

The variability in childcare regulations results in different physical activity requirements across the country. By providing high-level targets for physical activity recommendations, by way of provincial/territorial legislation, staff would have a baseline from which to begin supporting more active behaviors among the children in their care. Future research is needed to support translating physical activity policies into improved activity levels among young children in childcare and the role of screen-viewing in these venues.

## Background

Physical activity is vital to the healthy development of young children [1]. Likewise, limiting sedentary behaviors among this population, which includes minimizing excessive screen-viewing and sitting, is also linked to positive health outcomes [2]. In order to reap such benefits, the Canadian Society for Exercise Physiology (CSEP) released 24-Hour Movement Guidelines for the Early Years (0–4 Years) [3]. It is recommended that children 1 to 4 years engage in 180 min of physical activity each day (at any intensity), with at least 60 min of this time being spent in energetic play among 3–4-year-olds. Prolonged periods of sitting should be limited, with screen-time being avoided (under 2 years) or restricted to 1 hour per day (2–4 years). Despite the benefits of engaging in appropriate levels of each behavior, only 15% of young children have been reported to meet both the physical activity levels and sedentary behaviors guidelines, hence leaving much room for improvement [4].

The childcare environment has received much attention in recent years in relation to young children’s physical activity and sedentary behaviors [5–8]. Recent research purports that the childcare environment accounts for nearly 50% of the variation in young children’s physical activity levels [9, 10]; more influential than age, sex, and ethnicity. While many young children are enrolled in some form of non-parental care for extended periods of time each week [11–14], coupled with the lessons they learn regarding health behaviors (like nutrition, physical activity, and screen-viewing), the childcare environment is an ideal setting to target the activity behaviors of young children. Particularly from a health promotion perspective, it is important to target children early in life to ensure healthy habits are being formed and carried throughout the lifespan in an effort to limit chronic disease and support optimal health [15].

Unfortunately, the childcare setting has been repeatedly noted in the literature as an inactive and sedentary environment. Vanderloo et al. [5] and Temple et al. [16] reported that young children engaged in approximately 1.54 and 1.76 mins/h of moderate-to-vigorous physical activity (MVPA), respectively. Conversely, Tucker et al. [7] found that preschoolers in childcare spent 41.62 mins/h in sedentary behaviors. Brown and colleagues also reported that preschoolers in their study spent approximately 89% of their time in care engaged in sedentary behaviors [17]. Specific to screen-viewing (i.e., typically a proxy for sedentary behaviors among children), a recent systematic review highlighted the high prevalence of this behavior among young children in childcare [18]. Not surprisingly, efforts are warranted to improve this setting with regard to supporting healthy active behaviors among enrolled children.

Many factors or attributes within the childcare environment have been examined in relation to their impact on young children’s activity levels (e.g., childcare staff behaviors, equipment, space, etc.) [19–23]. In addition to these characteristics, outdoor play periods [24, 25] and active play [26] are oftentimes used as proxies for physical activity and are particularly noteworthy within the childcare environment given their positive relationship with this behavior. Specifically, in their review, Gray and colleagues noted that all included studies reported higher physical activity levels outside compared to inside and that children’s total physical activity was 2.2 to 3.3 times higher outdoors [25]. In addition, the presence of physical activity and sedentary behavior policies have been noted as a useful mechanism for childcare providers when structuring their daily programming and curriculum for enrolled children [27]. In a recent study exploring the physical activity and sedentary behavior policies present in New Zealand licensed childcare facilities (*n* = 237), Gerritsen and colleagues [28] identified that 35% of most centers had a physical activity policy in place, while none had a screen viewing policy. The noticeable lack of policy observed is concerning and warrants attention, as the impact of policies on activity behaviors is evolving. More specifically, preliminary results seem promising as O’Neill and colleagues found that the adoption of new physical activity standards in South Carolina childcare centers was associated with improvements in practices aimed at increasing children’s physical activity [29]. While advances have been made in Australia [30], the United States [20, 27, 29], and New Zealand [28], where physical activity and sedentary behavior (i.e., screen time) policies have been put in place in childcare centers, less work has been conducted in Canada.

In 2012, Vanderloo and colleagues undertook a review of all provincial and territorial childcare legislation to provide an overview of physical activity-specific regulations in Canada [31]. The result of that review highlighted the scarcity and variability in provincial and territorial policies pertaining to physical activity. In fact, only Ontario provided a specific time requirement in which young children should spend *outdoors* (though not necessarily indicative of physical activity time [31]). The Canadian childcare landscape has changed immensely in the past 5 years with the introduction of the Full-Day Kindergarten program in Ontario (which entails children who are age 4 start attending school full time – while before they would have attended school part-time, and still required childcare for the other part of the week) as well as voluntary accreditation standards for childcare facilities implemented in Alberta [32] and a revised standard of practice for British Columbia (which provide childcare facilities with standards above and beyond ministry requirements) [33]. Moreover, the 2012 review did not examine the presence of sedentary-specific regulations in provincial and territorial childcare Acts [31]. While some sedentary behaviors play an important role for young children’s development and learning (e.g., reading, colour, etc.), in childcare, concern and interest has primarily been with screen time. Given the recent interest and advancement in sedentary behavior research [34], specifically increased screen time among children [27, 35–39], consideration of both physical activity and screen-viewing regulations would provide researchers and public health officials with a clearer picture of how policy is (or is not) being used to encourage or support physical activity and limit sedentary behaviors in childcare.

Significant strides have been made in the physical activity and sedentary behavior literature for the early years. Concerted efforts have been undertaken to develop evidence-informed guidelines, understand the prevalence of physical activity among young children, and to identify factors within childcare facilities which support and deter these behaviors. What has been less purposeful, is consideration of the childcare policy landscape. Vanderloo et al.’s previous review is now 5 years old, did not explore sedentary behavior regulations, and recently, amendments have been undertaken to a number of provincial/territorial childcare legislations [31]. As such, an updated review is necessary. Therefore, the purpose of this paper was to provide an amended review of the legislative landscape regarding physical activity and sedentary behaviors (via screen-viewing) in childcare in Canada.

For the purposes of this review, the term legislation (oftentimes referred to as an Act), will be used, which represents written laws, whereas regulations describe the application and enforcement of the Act. Policies are documents or written statements/declarations that are used to interpret the regulation, and can transpire at the provincial/territorial level or be implemented at a local childcare level. Specific to the childcare environment in Canada, each province and territory has its own respective childcare Act and regulations (with the exception of Nunavut which adheres to the Northwest Territories’ documents). Based on these key documents, each center within the province/territory is mandated to follow, at a minimum, the actions set out within these regulations. Provincial/territorial legislation have the potential to help inform and enforce center-level policies in childcare, or at a minimum, serve as an adopted policy in these venues.

## Methods

A similar process to the 2012 review by Vanderloo and colleagues was undertaken [31]. Specifically, in February 2017, the individual childcare Acts and regulations for each province and territory in Canada were collected. As there is no national central database which housed these documents, the websites of each province’s and territory’s government were accessed to gather them. All retrieved legislation was then confirmed by two researchers as well as against the most recent copy of Canada’s Childcare Resource and Research Unit report [40]. Canadian experts in the field of physical activity and childcare were also contacted via email to ensure no salient policy documents from their respective provinces or territories were missed. Each province and territory (except Nunavut) had their own set of childcare acts and regulations.

Once all provincial and territorial Acts and regulations were retrieved, each document was read with a particular focus on any sections which spoke to child health, physical activity (e.g., active play, gross motor and large muscle play, physical fitness), screen time, and outdoor time. Given the length and detail of these documents, a keyword search (i.e., physical activity, sedentary behaviors, play, screen-time, TV, indoor space, outdoor space, development, motor, etc.) of each digital document was also undertaken to ensure no pertinent content was missed.

An extraction table was created to ensure a systematic process and consistent data retrieval as well as to facilitate comparisons across provinces and territories. Specifically, one researcher extracted the following data from each provincial and territorial document: province/territory name; name of Act and regulation(s); date of publication; age group of children covered by the legislation; presence and details of regulations focused on physical activity and/screen-time; details on playtime (indoors and outdoors); and, details on space infrastructure (indoors and outdoors). Given the level of this review (i.e., provincial/territorial Acts and regulations), no staff-specific information (as it pertains to physical activity training and development) was available for extraction. Extracted data was confirmed by a second researcher for accuracy. Once extracted, both researchers looked for common trends and themes across the provinces/territories.

## Results

### General observations

Of the 13 provinces and territories, 8 (62%) have updated or amended their childcare acts and regulations in the past 5 years. The types of facilities legislated under each act varied greatly across provinces and territories, in addition to the “classic” childcare center (i.e., daycare), other care providers were also mandated under these regulations, including home-based childcare, child minding, before and after school programs, and nursery schools. The age of the children covered under these acts and regulations also varied considerably from birth to 13 years, and up to 18 years for children with disabilities. The provision of before and after school care accounts for many of the older children (≥ 5 years) covered under the noted provincial and territorial legislations. See Table 1 for additional details.

**Table 1:** Provincial and territorial childcare legislation for physical activity and screen-viewing

Province/Territory	Name of Act and Regulation (Year)	Type of Facility Legislated	Age Groups	Provider: Child Ratios	PA Requirements	SV Requirements	Infrastructure – Indoors & Outdoors	Playtime – Indoors & Outdoors
Alberta	Child Care Licensing Act, 2008 Childcare Licencing Regulation 143/2008 (with amendments up to and including Alberta Regulation 152/2016)	Daycare centers	19 months to under 13 yrs	Under 12 months: 1:3 12–18 months: 1:4 19 months to 4.5 yrs: 1:8 Over 4.5 yrs: 1:10	•Must keep up with the physical, social, creative, intellectual, and emotional needs of the child ^≠^	N/A	•*Indoor play space:* ≥ 3.0m^2^ per child •*Outdoor play space:* ○ ≥ 2m^2^ for each child under 19 months ○ ≥ 4.5m^2^ for children over 19 months	Provide indoor and outdoor play materials
British Colombia	Community Care and Assisted Living Act, S.B.C. 2002, Chapter 75 Child Care Licensing Regulation 332/2007, including amendments to B.C. Reg. 178/2016 Child Care Subsidy Act, R.S.B.C. 1996, Chapter 26; Child Care Subsidy Regulation 74/97. Child Care BC Act, S.B.C. 2001. Chapter 4.	Group daycare centers Preschools Family childcare Child minding	0–5 yrs 30 months to 5 yrs 0–12 yrs 1.5–12 yrs	1:4 1:10 1:7 1:8	•Opportunities for social, emotional, physical and intellectual growth •Encourage development of large and small muscle skills	N/A	•*Indoor play space:* ≥ 3.7m^2^ per child *Outdoor play space*: ≥ 7m^2^ per child	•Provide periods for outdoor play (weather permitting)
Manitoba	Community Child Care Standards Act, C.C.S.M. c. C158. (amended 67/2016) Child Care Regulation, M.R. 62/86. Child Care Worker Retirement Benefits Regulation, M.R. 20/2011	Childcare centers Nursery school School-age childcare Family childcare homes Group childcare homes	0–12 yrs and 0–18 yrs for children with disabilities	12 wks to 1 yr: 1:3 1–2 yrs: 1:4 2–3 yrs: 1:6 3–4 yrs: 1:8 4–5 yrs: 1:9 5–6 yrs: 1:10 6–12 yrs: 1:15	•Play activities must allow for daily large and small muscle activity	N/A	•*Indoor play space:* ≥ 3.3m^2^ per child •*Outdoor play space:* ≥ 7m^2^	•Provide space and equipment for a variety of activities including fine motor activities •Provide time for outdoor play (weather permitting)^≠^
New Brunswick	Early Childhood Services Act. 2010. Family Services Act. 1980. Family Services Act and Day Care Regulations, 83–85, as amended.	Daycare centers Nursery Schools School-age childcare centers Community daycare homes	0–12 yrs	Birth to 24 months: 1:3 2 yrs: 1:5 3 yrs: 1:7 4 yrs: 1:10 5 yrs: 1:12 6–12 yrs: 1:15	N/A	•Television viewing is not part of the daily program	•*Indoor play space:* ≥ 3.25m^2^ per child •*Outdoor play space:* ≥ 4.5m^2^ per child	•Provide time for outdoor play (weather permitting)^≠^ •Provide equipment that promotes gross and fine motor movement
Newfoundland & Labrador	Child Care Services Act. — SNL 1998, chapter c-11.1, amended 1999 c22 s6, 2001 c36. Child Care Services Regulation 37/99, revised March 2007.	Childcare centers School-age childcare centers Family childcare	0–13 yrs	0–24 months: 1:3 2-3 yrs: 1:5 3–5.75 yrs: 1:8 4.75–7 yrs: 1:12 7–12.9 yrs 1:15	N/A	N/A	•*Indoor play space:* ≥ 3.3m^2^ per child	•Must have access to an outdoor play area if in care for 4 + hrs •Outdoor play areas not required for family/ home daycare
Northwest Territories	Child Day Care Act and the Child Day Care Standards and Regulations 1988 (2013). Child Day Care Standards and Regulations, R-053-2012, with amendments R-092-2014	Center daycare facilities Preschool daycare Out of school daycare Family home daycare	0–11 yrs	0–12 months: 1:3 13–24 months: 1:4 25–35 months: 1:6 3 yrs: 1:8 4 yrs: 1:9 5–11 yrs: 1:10	•Facilitate and stimulate physical development •Opportunity to participate in activities that promote physical fitness for 30 mins/day	N/A	•*Indoor play space:* ≥ 2.75 m^2^ per child •*Outdoor play space:* ≥ 5m^2^ per child	•Provide daily outdoor play activities for each child^≠^ •Space should be developmentally appropriate for children
Nova Scotia	Day Care Act. Chapter 120, of the Revised Statutes, 1989. R.S., c. 120. s. 1.41 Day Care Regulations Reg 193/2010, N.S. Reg. 226/2014 and including O.I.C. 2014–531 (December 22, 2014), N.S. Reg. 227/2014	Childcare centers Family daycare homes	0–12 yrs	*Childcare centers* Infant: 1:4 Toddler: 1:6 Preschooler: 1:8 *Family Daycare Homes* Infants: 1:3 School age: 1:8	•Provide opportunities for physical activity •Activities must stimulate physical development	N/A	•*Indoor play space:* ≥ 2.75m^2^ per child •*Outdoor play space*: ≥ 7m^2^ per child	•Provide outdoor playtime in the morning and afternoon (weather permitting)
Nunavut^±^	Child Day Care Act and the Child Day Care Standards and Regulations 1988 (2013). Child Day Care Standards and Regulations, R-053-2012, with amendments R-092-2014	Center daycare facilities Preschool daycare Out of school daycare Family home daycare	0–11 yrs	0–12 months: 1:3 12–24 months: 1:4 25–35 months: 1:6 3 yrs: 1:8 4 yrs: 1:9 5–11 yrs: 1:10	•Facilitate and stimulate physical development •Opportunity to participate in activities that promote physical fitness for 30 mins/day	N/A	•*Indoor play space:* ≥ 2.75 m^2^ per child •*Outdoor play space*: ≥ 5m^2^ per child	•Provide daily outdoor play activities for each child^≠^ •Space should be developmentally appropriate for children
Ontario	Child Care and Early Years Act, 2014, S.O. 2014, c. 11, Sched. 1 Ontario Regulation 137/15: GENERAL	Childcare centers Licensed home childcare	0–12 yrs and 0–18 yrs for children with disabilities	*Indoors* Infants: 3:10 Toddlers: 1:5 Preschooler: 1:8 Kindergarten: 1 to 13 *Outdoors* No reduced ratios	•Activities that promote gross and fine motor skills development •Incorporate active and quiet play into daily programming	N/A	•*Indoor play space:* ≥ 2.8m^2^ per child •*Outdoor play space:* ≥ 5.6m^2^ per child	•Provide 2 h of outdoor playtime for every 6+ hrs in care (weather permitting) •Incorporate indoor and outdoor play into daily programming
Prince Edward Island	Child Care Facilities Act. 1988. Child Care Facilities Regulations. 1988. The Social Assistance Act. 2003 (Dept of Community Services and Seniors)	Early childhood center Family childcare School-aged childcare center	Under 12 yrs	*Indoors* Under 22 months: 1:3 22 months to 3 yrs: 1:5 Over 3 yrs: 1:10 School-age: 1:15 *Outdoors* Under 22 months: 1:3 22 months to 3 yrs: 1:7 Over 3 yrs: 1:15 School-age: 1:22	•Activities designed to enhance emotional, social, cognitive and gross and fine motor development •Opportunities for active and quiet play	N/A	•*Indoor play space:* ≥ 3.5m^2^ per child •*Outdoor play space:* ≥ 7m^2^ per child	•Provide outdoor play opportunities^≠^
Quebec	*Ministère de la Famille:* Educational Childcare Act (R.S.Q., chapter S-4.1.1). Educational Childcare Regulation chapter S-4.1.1, r. 2 *Ministère de l’Éducation, de l’Enseignement Supérieur et de la recherche:* Règlement sur les services de garde en milieu scolaire. L.R.Q., c.I-13.3., a. 454.1; 1997, c.58, a.51; 1997, c.96, a.132.	Childcare center Daycare center Home or Family childcare School-age childcare	0–14 yrs	< 18 months: 1:5 18 months to < 4 yrs: 1:8 4 yrs to < 5 yrs: 1:10 > 5 yrs: 1:20	•Provide educational activities that promote physical development •Programs which foster emotional, social, moral, cognitive, language, physical, and motor development^≠^	N/A	•*Indoor play space:* ○ ≥ 4m^2^ per child under 18 months ○ ≥ 2.75m^2^ per child over 18 months •*Outdoor play space:* ≥ 4m^2^ per child	•Provide daily outdoor play periods (weather permitting)^≠^ •Play equipment must meet Canadian safety standards
Saskatchewan	Child Care Act. Bill 8, 1990 as amended by the Statutes of Saskatchewan, 2000 Child Care Regulations, Chapter C-7.31 Reg 1 (effective June 15, 2015) as amended by Saskatchewan Regulations 69/2015 and 49/2016.	Childcare centers School-age childcare Family childcare homes	0–13 yrs and 0–15 yrs for children with disabilities	1:15	N/A	N/A	•*Indoor play space:* ○≥ 3.7m^2^ per infant ○≥ 3.25m^2^ per toddler, preschool, and school-aged child •*Outdoor play space*: ≥ 7m^2^ per child	•Provide materials for indoor and outdoor activities •Provide outdoor play periods
Yukon	Child Care Act, 1990/087 Child Care Center Program Regulations, 1995 Family Day Home Program Regulation, 1995 School-Age Program Regulation, 1995 Child Care Subsidy Regulation, 1995	Childcare centers School-age childcare Family day home	12 yrs and under and 16 yrs and younger for children with disabilities	Infants to 18 months: 1:4 18 months to 3 yrs: 1:6 3–5 yrs: 1:8 School-age: 1:12	•Offer physical activities to promote large muscle development and physical competences (e.g., running and climbing) •Promote small muscle development and physical well-being •Provide opportunities for vigorous activities	N/A	•*Indoor play space:* ≥ 4m^2^ per child •*Outdoor play space:* ≥ 5m^2^ per child	•Opportunity to play outdoors everyday (weather permitting)^≠^ •Outdoor equipment must be provided for large muscle activity

*PA* physical activity, *SV* screen-viewing, ^*±*^follows same legislation as Northwest Territories, ^≠^specific amount of time not specified. The childcare Acts and regulations for each province and territory are presented to provide information on the governing laws as well as any related prescriptive and enforcement documents

### Physical activity requirements

Only 3 provinces (Northwest Territories, Nunavut, and Nova Scotia) explicitly mentioned physical activity in their regulations. Of these, Northwest Territories and Nunavut (who are both covered by Northwest Territories’ legislation) provided a specific amount of time required for children to participate in activities that promote physical fitness (30 min/day). For those provinces/territories that had no specific time requirements for physical activity within their daily programming, some did mandate ensuring children were provided with opportunities to engage in “active” (Ontario, Prince Edward Island) or “vigorous” (Yukon) play or activities that develop large muscle or gross motor skills (British Colombia, Manitoba, New Brunswick, Ontario, Prince Edward Island, Quebec, Yukon). Consequently, all provinces provide *general recommendations* to afford gross motor movement; but the majority give no specific requirements for how much, how, or at what intensity.

#### Indoors and outdoors playtime periods

All provinces’ and territories’ required periods of daily outdoor play pending appropriate weather conditions. Two provinces’ recommendations go further than simply mandating daily outdoor time. Specifically, the policy in Nova Scotia requires that children need to be provided with 2 outdoor play periods per day, while Ontario specifically states that for every 6 h in care, children should be provided with 2 h of outdoor play (1 h in the morning and 1 h in the afternoon).

### Screen-viewing requirements

Only 1 out of the 13 provinces and territories made mention of screen-viewing. Specifically, New Brunswick’s regulations state that television viewing should not be a part of the children’s daily programming during care hours. All other provinces and territories provide no guidance regarding the use and number of screens, including computers, television, and tablets.

### Space infrastructure – Indoors and outdoors

The provincial and territorial regulations have specific indoor infrastructure requirements, but the amount of indoor space allotted to each child varied (2.75 to 3.7 m^2^). Quebec and Saskatchewan specified smaller space allocations for younger children indoors. While safety was always mentioned when discussing the quality of indoor space, few provinces or territories made mention of the promotion of physical activity or gross motor movement indoors. Specifically, Alberta’s and Saskatchewan’s regulations stated that materials for indoor play must be provided, Manitoba’s and Yukon’s stated that space and equipment to engage in a variety of gross and fine motor activities need to be provided, and Quebec’s required that the play equipment must meet Canadian Safety Standards (CSA).

Outdoor space allotment per child also varied across provinces and territories ranging from 2 to 7m^2^. Alberta, New Brunswick, and Quebec specified smaller space allocations for children outdoors, while British Colombia, Manitoba, Nova Scotia, Prince Edward Island, and Saskatchewan have the largest space requirements. Newfoundland and Labrador did not provide any outdoor space allocations. Some provinces/territories also provided different space provisions for children based on age. For example, Alberta required more than 2m^2^ of outdoor space per child under 19 months, and more than 4.5m^2^ per child over 19 months. Amount of indoor space allotted per child also differed based on age for Quebec and Saskatchewan.

## Discussion

The purpose of this paper was to conduct an updated review of the current provincial and territorial childcare legislation in Canada as it pertains to physical activity and sedentary behaviors (via screen-viewing). Given recent (albeit modest) advances in childcare polices in the last 5 years, the release of Canadian 24-Hour Movement Guidelines for the Early Years (0–4 Years), and the surge of interest in sedentary behaviors (specifically screen-viewing) among young children, an up-to-date examination of the childcare legislation landscape was necessary. These data shed light on the limited and inconsistent mandate of physical activity and screen-viewing policies in childcare centers across Canada. One reason attributing to this lack of policy support within the childcare setting, may in large be due to the common misconception that young children are very active (and not at-risk for adverse consequences), and therefore, this behavior is not viewed as a priority among policymakers. Interestingly, given the sedentariness of the childcare environment [7], coupled with the impact provincial/territorial-level regulations can have on healthful behaviors [27], lack of policy may represent a missed opportunity. For example, a study by Carson et al. examined the impact of Alberta’s new child care accreditation standards (which included specific requirements regarding physical activity and sedentary behaviors) on enrolled children’s activity levels [41]. Over a 6.5-month period, small decreases in sedentary behaviors among toddlers (3.1 min/h) were noted, as well as small increases in MVPA among this population (1.7 min/h). This evidence demonstrates how the introduction of more specific policies (or regulations) could have a positive impact on activity behaviors in childcare.

This work extends Vanderloo and Tucker’s [31] previous exploration to provide an up-to-date look at the childcare legislation in Canada. A number of childcare Acts have been updated since the 2012 review (8 of 13). Despite the revisions that have transpired, very few changes were observed with regard to physical activity requirements. In light of the recent release of Canada’s 24-Hour Movement Guidelines for the Early Years [3] guidelines specific to the early years, future updates to provincial and territorial requirements for childcare should reflect these recommendations. These guidelines require young children to engage in 180 min of physical activity (any intensity) per day, with preschoolers focusing on the accumulation of 60 min of energetic play as well [42]. Based on the notion that many children spend a substantial portion of their day in childcare [13], if you consider these guidelines in the context of a typical 8-h childcare day, these children should be afforded approximately 120 min of physical activity daily (with 40 min of energetic play). In the absence of such specific legislation across Canada, it may make the attainment of these guidelines challenging for young children. While provincial and territorial Ministries may be trying to provide general guidance so as to not restrict childcare centers in the programming and implementation, in the absence of specific requirements; however, there also affords the opportunity for a great deal of variability, and low levels of activity, if it is not a priority of the childcare staff [43]. Although voluntary and/or optional to implement, Quebec [44], Alberta [32], and British Columbia [33] have recently released resources which provide more detailed guidance regarding physical activity, sedentary behaviors, and screen-viewing in childcare. For example, Quebec has created a reference manual to be used by childcare staff which provides additional guidance regarding nutrition, active play, and sedentary time [44]. In late 2017, British Columbia also has plans to enforce a new standards document focused on active play in childcare [33]. Nevertheless, the lack of specific regulation within the provincial and territorial Acts was somewhat surprising given the major health implications of leaving these behaviors unaddressed[2] as well as the ongoing research efforts to change these behaviors. Opportunities exist for improved standards of care in line with the evidence-informed 24-Hour Movement Guidelines[3] to ensure all children are receiving appropriate physical activity affordances (and minimized sedentary pursuits).

This work, unlike Vanderloo et al.’s previous provincial/territorial review [31], explored screen time regulations. A recent systematic review not only reported screen-viewing to be moderately high among this young sample in childcare, but also noted that this particular environment is conducive to this form of sedentary behavior [18]. It was interesting to note that only one province (i.e., New Brunswick) provided regulations regarding screen time. This may be a consequence of the use of screens within childcare centers for education purposes (e.g., computers, iPads, etc.), or could be a consequence of childcare regulations not yet being ready to align with the Canadian recommendations. Nevertheless, given the negative health consequences associated with excessive screen use (e.g., obesity, high blood pressure, irregular sleep patterns, behavioral issues [45–47]), it is important that childcare centers make purposeful and educational use of such devices, and limit exposure. Given that many young children have access to and use screens at home, limiting use to less than the Canadian recommendations in the childcare environment, would be ideal.

Research exploring the direct relationship between physical activity and sedentary time policies and their impact on preschoolers’ activity behaviors is in its infancy. However, recent research in the United States shows promise, as O’Neill and colleagues reported that new physical activity standards in South Carolina childcare centers were associated with improvements in practices aimed at increasing children’s physical activity [29]. Despite this, the impact of these policies on preschoolers’ actual physical activity levels was not explored, and therefore, additional research is needed to better understand the potential role of policies on activity behaviors. Moreover, from a health promotion perspective, the addition of these policies makes good sense, given that they impact all children in all centers captured under that regulation. What is less clear, is how the implementation of them, by individual centers and childcare staff influences their effectiveness. As such, additional research in this area is necessary, given that childcare staff have been shown to have a strong influence on the activity behaviors of the children for which they care [5]. This is also important to consider because early childhood education students who are active are more likely to feel confident to engage preschoolers in physical activity; [43] therefore, it is quite possible that even with specific state or provincial level policies in place, implementation of said policies will vary greatly. This seems possible, given Erinosho et al.’s recent study which displayed that policies about staff supervision of screen use were negatively associated with screen time, but counterintuitively, policies about physical activity were associated with less time in physical activity [27]. Again, highlighting that the creation of a policy alone, may not support appropriate physical activity participation, and guidance and resources on the best mechanism to achieve those recommendations may be warranted to improve adoption and implementation success. Consequently, given the degree of implementation of said policies may influence physical activity levels, it is therefore important that the policy be feasible, and in line with current early childhood programming.

Childcare provider to child ratios and space requirements were also extracted for examination in relation to young children’s physical activity during childcare hours. Such information not only provides clear description of the number of childcare providers/children grouped into one class, but in maintaining strict alignment to these regulations, could have an impact on how much space is available for active and gross motor play (indoors and outdoors). In fact, tighter ratios (which can fluctuate throughout the day) and space allocations may be more conducive to quiet activities, which mainly involve sitting and/or little movement. Additionally, transitions and reductions in staff over breaks and at the beginning/end of day may influence the number of opportunities available for outdoor play.

All provincial and territorial regulations required that children must be provided with daily outdoor play opportunities during favorable weather conditions, and most did not specify time requirements. It is quite possible that these outdoor sessions are intended to serve as a proxy for physical activity; however, given that children are the most active during the first 10 min and then their activity levels decline [48–50], it may be necessary to revisit scheduling of outdoor playtime in these settings. Research suggests that young children are more active outdoors than indoors [24], and the space requirements in these facilities are more conducive to gross motor movement, therefore, outdoor play is an important variable for consideration with this group. In a recent study conducted by Tucker and colleagues [51], their physical activity intervention included the modification of outdoor playtime in childcare centers – revising the two, 60-min sessions required in Ontario to four, 30-min sessions. After the 8-week intervention, these researchers noted improved physical activity levels and reduced sedentary time among the children who received this intervention; however, the effects were not sustained 6- and 12-months post-intervention. The findings of Tucker et al.’s work highlight not only the importance of outdoor play for young children, but how the frequency and duration of outdoor play in childcare centers is also important. Additionally, activity levels have been documented to be higher during warmer weather; [52, 53] therefore, efforts may be necessary to ensure children are being dressed appropriately for outdoor play during all weather conditions and seasons, including rain and snow, so that they do not miss out on important physical activity opportunities (exception: extreme heat advisory or freezing conditions).

### Implications on the international stage

Though the objective and findings of this work focused on the Canadian context, there are key learnings that can be drawn for international audiences. Similar research has been undertaken in other countries. For example, Benjamin and colleagues [54] in the United States undertook a comparable study examining the presence of state-level legislation in relation to obesity prevention and physical activity promotion. Just like in Canada, such policy-level support was missing in most states, and in states where such legislation was present, there was quite a bit of variability. In New Zealand, Gerritsen et al. [28], noted that only 35% of childcare facilities had a written physical activity policy, with none devoted to screen-based restrictions. Collectively, the findings from this body work underscore the importance of additional research devoted to exploring the viability and utility of policy (be it at the province/state level or at the organization level) as a mechanism for providing standardized care devoted to supporting physical activity levels within the childcare environment that are in line with national recommendations [3, 55].

### Strengths and limitations

The current paper provides an up-to-date look at the childcare legislation landscape in Canada, in light of recent changes to provincial regulations and the addition of Canadian physical activity and sedentary behavior guidelines. However, limitations must be mentioned. Specifically, although some provinces and territories require physical activity programming, this paper did not examine the quality and implementation of said programming. Future research would benefit from considering the impact of physical activity and screen viewing policies on each respective behavior, along with the quality of the programming that is implemented to meet these provincial requirements. Moreover, research examining how the current childcare regulations are enforced in each province/territory and whether some measure of compliance with regard to the legislation exists within the childcare setting is warranted. Lastly, the applicability of these findings to the childcare setting in other countries may be limited as the structure of Canadian provinces/territories and the implementation of childcare legislation therein may vary.

## Conclusion

Each province and territory provides its own legislation regulating the programming within center-based childcare centers (and some home-based facilities). Only a few provinces/territories provide specific guidance regarding the amount and frequency of physical activity for children attending childcare. The variability between provinces and territories results in different physical activity requirements across the country. This is an important finding given that young children, regardless of the province or territory in which they reside, are recommended to engage in 180 min of physical activity each day (with preschoolers also engaging in 60 min of energetic play) [3]. The policies in some provinces/territories may be doing a better job in supporting these behaviors. Alternatively, the onus is on the individual childcare center to generate and implement physical activity curriculum. By issuing provincial/territorial legislation for physical activity and screen-viewing, staff will at least be provided with a baseline or minimum standard of care, from which they can start to encourage physical activity (and minimize screen time) among the children in their care. Once in place, additional steps can be taken to ensure the legislation is being followed and enforced. While the authors acknowledge that the mere development of such regulations may have a more distal and less direct path to the children within these centers, given the low levels of physical activity and the high sedentary time reported in Canadian childcare [5, 7, 16], setting-specific regulations represent one way to mandate childcare facilities to put physical activity at the forefront of providing good care for young children. Following this step, resources must be allocated to assist childcare organizations with implementation of such policies. The ratification or amendments of these regulations may be a missed opportunity to prioritize physical activity (and appropriate screen-viewing) among young children in childcare, attention is warranted to prioritize this on the political agenda in Canada. Future research is needed to support translating physical activity policies into increased activity levels among young children in childcare.

## References

[1] Timmons BW, Naylor PJ, Pfeiffer KA (2007). Physical activity for preschool children - how much and how? Can. Aust J Public Health.

[2] Leblanc AG, Spence JC, Carson V (2012). Systematic review of sedentary behaviour and health indicators in the early years (aged 0-4 years). Appl Physiol Nutr Metab.

[3] Tremblay MS, Chaput JP, Adamo KB, Aubert S, Barnes JD, Choquette L (2017). Canadian 24-hour movement guidelines for the early years (0-4 years): an integration of physical activity, sedentary behaviour, and sleep. BMC Public Health.

[4] Garriguet D, Carson V, Colley RC, Janssen I, Timmons BW, Tremblay MS (2016). Physical activity and sedentary behjaviour of Canadian children aged 3 to 5. Health reports [catalogue no. 82–003-X].

[5] Vanderloo LM, Tucker P, Johnson AM, Van Zandvoort MM, Burke SM, Irwin JD (2014). The iInfluence of Centre-based childcare on preschoolers' physical activity levels: a cross-sectional study. Int J Environ Res Public Health.

[6] Hinkley T, Salmon J, Okely AD, Hesketh K, Crawford D (2012). Correlates of preschool children's physical activity. Am J Prev Med.

[7] Tucker P, Vanderloo LM, Burke SM, Irwin JD, Johnson AM (2015). Prevalence and influences of preschoolers' sedentary behaviors in early learning centers: a cross-sectional study. BMC Pediatr.

[8] Ward DS, Benjamin SE, Ammerman AS, Ball SC, Neelon BH, Bangdiwala SI (2008). Nutrition and physical activity in child care: results from an environmental intervention. Am J Prev Med.

[9] Pate RR, Pfeiffer KA, Trost SG, Ziegler P, Dowda M (2004). Physical activity among children attending preschools. Pediatrics.

[10] Finn K, Johannsen N, Specker B (2002). Factors associated with physical activity in preschool children. J Pediatr.

[11] Cleveland G, Forer B, Hyatt D, Japel C, Krashinsky M (2008). New evidence about child care in Canada: use patterns, affordability and quality. Inst Res Public Policies.

[12] Sinha M (2014). Spotlight on Canadians: results from the general social survey: child care in Canada.

[13] Bushnik T. Child Care in Canada. 1–00. 2006. Statistics Canada. Ottawa: Children and Youth Research Paper Series.

[14] Organisation for Economic Co-operation and Development (2017). Education at a glance 2017: OECD indicators.

[15] Goldfield GS, Harvey A, Grattan K, Adamo KB (2012). Physical activity promotion in the preschool years: a critical period to intervene. Int J Environ Res Public Health.

[16] Temple VA, Naylor PJ, Rhodes RE, Wharf Higgins J (2009). Physical activity of children in family child care. Appl Physiol Nutr Metab.

[17] Brown WH, Pfeiffer KA, McIver KL, Dowda M, Addy CL, Pate RR (2009). Social and environmental factors associated with preschoolers' nonsedentary physical activity. Child Dev.

[18] Vanderloo LM. Screen-viewing among preschoolers in childcare: a systematic review. BMC Pediatr. 2014;14:205. 10.1186/1471-2431-14-205 PMC4149526 25129567

[19] Vanderloo LM, Tucker P, Johnson AM, Burke SM, Irwin JD (2015). Environmental influences on preschoolers' physical activity levels in various early learning facilities. Res Q Exerc Sport.

[20] Dowda M, Brown WH, McIver KL, Pfeiffer KA, O'Neill JR, Addy CL (2009). Policies and characteristics of the preschool environment and physical activity of young children. Pediatrics.

[21] Trost SG, Ward DS, Senso M (2010). Effects of child care policy and environment on physical activity. Med Sci Sports Exerc.

[22] Hinkley T, Salmon J, Crawford D, Okely AD, Hesketh KD (2016). Preschool and childcare center characteristics associated with children's physical activity during care hours: an observational study. Int J Behav Nutr Phys Act.

[23] Gubbels J, Van Kann DHH, Jansen MWJ. Play equipment, physical activity opportunities, and children's activity levels at childcare. J Environmental Pub Health. 2012;2012:8. 10.1155/2012/326520 PMC3395256 22811736

[24] Vanderloo LM, Tucker P, Johnson AM, Holmes JD (2013). Physical activity among preschoolers during indoor and outdoor childcare play periods. Appl Physiol Nutr Metab.

[25] Gray C, Gibbons R, Larouche R, Sandseter EBH, Bienenstock A, Brussoni M (2015). What is the relationship between outdoor time and physical activity, sedentary behaviour, and physical fitness in children? A systematic review. Int J Environ Res Public Health.

[26] Truelove S, Vanderloo L, Tucker P (2016). Defining and measuring active play among young children: a systematic review. J Phys Act Health.

[27] Erinosho T, Hales D, Vaughn A, Mazzucca S, WARD DS (2016). Impact of policies on physical activity and screen time practices in 50 child-care centers in North Carolina. J Phys Act Health.

[28] Gerritsen S, Morton SMB, Wall CR (2016). Physical activity and screen use policy and practices in childcare: results from a survey of early childhood education services in New Zealand. Aust N Z J Public Health.

[29] O'Neill JR, Dowda M, Benjamin Neelon SE, Neelon B, Pate RR (2017). Effects of a new state policy on physical activity practices in child care centers in South Carolina. Am J Public Health.

[30] Wolfenden L, Neve M, Farrell L (2011). Physical activity policies and practices of childcare centres in Australia. J Paediatr Child Health.

[31] Vanderloo LM, Tucker P, Ismail A, Van Zandvoort MM (2012). Physical activity opportunities in Canadian childcare facilities: a provincial/territorial review of legislation. J Phys Act Health.

[32] Alberta Government (2017). Alberta child care accreditation standards.

[33] British Columbia Government (2017). Director of licensing standard of practice - active play.

[34] Sedentary Behaviour Research Network (2012). Standardized use of the terms "sedentary" and "sedentary behaviours". Appl Physiol Nutr Metab.

[35] He M, Irwin JD, Sangster Bouck LM, Tucker P, Pollett GL (2005). Preschoolers' screen viewing behaviors among preschoolers: Parents' perceptions. Am J Prev Med.

[36] De Decker E, De Craemer M, De Bourdeaudhuij I, Wijndaele K, Duvinage K, Koletzo B (2013). Influencing factors of screen time in preschool children: an exploration of parents' perceptions through focus groups in six european countries. Obes Rev.

[37] Hinkley T, Salmon J, Okely AD, Crawford D, Hesketh K (2012). Preschoolers' physical activity, screen time, and compliance with recommendations. Med Sci Sports Exerc.

[38] Hoyos Cilero I, Jago R (2010). Systematic review of correlates among screen-viewing among young children. Prev Med.

[39] Canadian Paediatric Society, Digital Health Task Force. Screen time and young children: Promoting health and development in a digital world. Paediatrics & Child Health. 2017;22(8):461–68. 10.1093/pch/pxx123 PMC5823000 29601064

[40] Childcare Resource and Research Unit (2008). Early childhood education and Care in Canada.

[41] Carson V, Clark D, Ogden N, Harber V, Kuzik N (2015). Short-term influence of revised provincial accreditation standards on physical activity, sedentary behavior, and weight status in Alberta, Canada child care centers. Early Childhood Educ J.

[42] Candian Society of Exercise Physiology (2012). Canadian physical activity guidelines for the early years: 0–4 years.

[43] Martyniuk O, Tucker P (2014). An exploration of early childhood education students' knowledge and preparation to facilitate physical activity for preschoolers: a cross-sectional study. BMC Public Health.

[44] Ministere de la Famille (2017). Gazelle et Potiron : Quebec.

[45] Shea S, Basch CE, Gutin B (1994). The rate of increase in blood pressure in children 5 years of age is related to changes in aerobic fitness and body mass index. Pediatrics.

[46] Paik H, Comstock G (1994). The effects of television violence on antisocial behaviour: a meta-analysis. Community Res.

[47] Thompson DA, Christakis DA (2005). The association between television viewing and irregular sleep schedules among children less than 3 years of age. Pediatrics.

[48] Pate RR, Dowda M, Brown WH, Mitchell J, Addy C (2013). Physical activity in preschool children with the transition to outdoors. J Phys Act Health.

[49] McKenzie TL, Sallis JF, Elder JP (1997). Physical activity levels and prompts in young children at recess: a two-year study of a bi-ethnic sample. Res Q Exer Sport.

[50] Greever CJ, Sirard J, Alhassan S (2015). Objective analysis of preschoolers' physical activity patterns during free playtime. J Phys Act Health.

[51] Tucker P, Vanderloo LM, Burke SM (2017). Impact of the supporting physical activity in the childcare environment (SPACE) intervention on Preschoolers' physical activity levels and sedentary time: a cluster randomized controlled trial. Int J Environ Res Public Health.

[52] Tucker P, Gilliland J (2007). The effect of season and weather on physical activity: a systematic review. Pub Health.

[53] Carson V, Spence JC, Cutumisu N, Boule N, Edwards J (2010). Seasonal variation in physical activity among preschool children in a northern Canadian city. Res Q Exer Sport.

[54] Benjamin SE, Cradock A, Walker EM, Slining M, Gillman MW (2008). Obesity prevention in child care: a review of U.S. state regulations. BMC Public Health.

[55] Okely A, Ghersi D, Hesketh K, Santos R, Loughran SP, Cliff DP (2017). A collaborative approach to adopting/adapting guidelines - the Australian 24-hour movement guidelines for the early years (birth to 5 years): an integration of physical activity, sedentary behavior, and sleep. BMC Public Health.

